# Sound and Silence: The Effects of Environmental Conditions on State Boredom in an Online Study during the COVID-19 Pandemic

**DOI:** 10.3390/bs12080282

**Published:** 2022-08-12

**Authors:** Alana J. Anderson, Claire E. McMeen, Sammy Perone, Elizabeth H. Weybright

**Affiliations:** 1 Department of Psychiatry, Washington University School of Medicine, Saint Louis, MO 63108, USA; 2 Department of Human Development, Washington State University, Pullman, WA 99164, USA

**Keywords:** boredom, attention, distraction, multitasking

## Abstract

Boredom is a negative emotion commonly experienced in mundane situations. Boredom is thought to arise from a mismatch between individuals and their expectation for environmental stimulation. People attempt to reduce boredom by increasing the stimulation in their environment (e.g., turning on TV or music). Theories of boredom suggest external stimulation may cue the individual to expect more stimulation than the mundane task offers—thereby increasing boredom. Researchers adapted lab-based tasks to online during the COVID-19 pandemic, which allowed participants to set the study’s environmental conditions. Our method involved data collected online during the COVID-19 pandemic. We tested whether 137 college-age participants who reported being alone in a noisy room experienced more boredom after a mundane task than those who were alone in a quiet room. Results showed individuals in a noisier environment reported more boredom following a repetitive task than those in a quieter environment. Some people, high in trait boredom, experience boredom more frequently or cannot tolerate it. Our results revealed that the effects of environmental condition remained after controlling for the influence of trait boredom. In the discussion, we describe links to extant boredom research and implications for researchers collecting data online and individuals attempting to mitigate boredom.

## 1. Introduction

Boredom is a negative emotion common in daily life. Even a brief experience with boredom can reduce feelings of meaningfulness and motivation [1], increase aggressive tendencies [2], and lead to risk taking [3]. Boredom is associated with both low arousal states (e.g., fatigue) as well as high arousal states (e.g., agitation or anxiety) [4,5] and is often experienced in mundane tasks that constrain an individual’s autonomy. For example, most people experience boredom at work or school, or when waiting in airports or medical offices [6]. When people need to complete mundane tasks, such as homework, they may change their environment to reduce boredom by adding stimulation, such as turning on the TV, studying with friends, or listening to music. Studies have shown too much stimulation places demands on attention that exceed people’s capacity, increasing boredom [7]. Thus, the choice to add stimulation to reduce boredom may instead increase it. During the COVID-19 pandemic, many researchers were forced to adapt lab-based studies for online data collection due to safety measures in place that prohibited face-to-face research protocols to reduce the spread of the virus. Unlike controlled lab settings, online data collection allows participants to choose their environment, just as they might when completing tasks for school or work at home. In the current study, we tested whether people who completed a mundane task as part of an online study in a noisy environment experienced more boredom than people who completed the task in a quiet environment. Addressing this question may be especially relevant to typical college-age students because prior studies have shown they are likely to turn on music or use social media to help pass time while studying or working [8].

Theories of boredom posit that boredom arises when people are not in an attentionally engaging or meaningful activity [9,10], signaling a need to shift attention to a more satisfying activity [11]. For example, the Boredom Feedback Model (BFM) posits that boredom arises when attentional engagement is inadequate, leading attention to be cast internally, externally, or back to the task in an attempt to resolve the differences between the desired level and actual levels of attentional engagement [7,9]. Whether people experience boredom has been shown to be influenced by the match between attentional engagement and stimulation present in the environment. According to the Meaning and Attention Components (MAC) model, too little stimulation is not attentionally engaging and too much stimulation taxes attention, both of which lead to boredom [7]. Expectations about how engaging an environment might be can also influence boredom. According to the Dual-Self Model of boredom [10], when cues indicate the environment will be engaging, expectations are high. However, if those expectations go unmet, boredom ensues. For example, people report independent work is more boring when surrounded by coworkers than when they work alone [12], presumably because the presence of people sets an expectation for social engagement, which goes unmet, leading to high levels of boredom. The presence of alternative activities might also influence boredom. For example, opportunity cost models posit that when the presence of a more optimally engaging alternative is present, the cost of allocating resources to complete a less engaging task induces unpleasant feelings, such as boredom, which signal a need to shift toward a more engaging alternative [10].

In the current study, we examined environmental influences on boredom while completing a virtual peg turning task as part of an online study. The peg turning task has been used in prior studies and shown to induce high levels of boredom relative to other tasks [13]. Originally, our study was designed to test whether priming participants to apply coping strategies while completing the task in the lab would reduce state boredom. The study was adapted for an online platform at the beginning of the COVID-19 pandemic. No effect of priming was observed. However, we purposefully asked participants to report information about their environment when completing the study because we were concerned about the possible role of outside influences not typical in lab studies. These questions asked if they were alone and in a quiet environment or alone and in a noisy environment, such as listening to music or TV. This enabled us to test whether these different environmental conditions influenced state boredom.

We do not know the reasons why some people completed the peg turning task in a noisy environment. However, the MAC, Dual-Self, and opportunity cost models of boredom all led us to expect people who completed the peg turning task in a noisy environment to experience more boredom than those who completed it in a quiet environment. The MAC model posits that more stimulation increases attentional demands, leading to more boredom [7,10]. We reasoned that people who chose to complete the peg turning task in a noisier environment would have more demands on their attention and experience more boredom. The Dual-Self model posits that high expectations for engagement that go unmet should result in more boredom [10]. The opportunity cost models posit that the presence of a more engaging alternative, such as listening to music or TV, should induce higher levels of boredom as a signal to shift to engage in a more satisfactory activity [10,14]. 

The focal point of the current study was on state boredom. However, some people experience boredom on a trait level. There are two types of trait boredom: boredom proneness, which reflects the tendency to experience boredom in daily life, and boredom susceptibility, which reflects an aversion to lack of novelty [15]. Since people high in trait boredom may seek, either intentionally or inadvertently, a more or less stimulating environment, we controlled for the influence of trait boredom on the effects of environmental condition. The conditions under which this study was conducted are not unlike those commonly experienced in daily life. For example, people are often required to complete mundane school or work tasks and often create environments to make completing the task more enjoyable, such as playing music, watching TV, or sitting with friends [8]. However, doing so may lead to especially high levels of boredom.

## 2. Materials and Methods

### 2.1. Participants 

The final sample consisted of 137 undergraduate college students at one public 4-year institution aged 18–22 years (*M_age_* = 19.93 years, *SD_age_* = 1.13 years, 116 females, 21 males). Participants were recruited from university courses in psychology and human development departments and earned extra credit for the completion of the study. Instructors of the courses were given announcements and students used a link to access the online study. This sample was selected from a larger sample with participants ranging in age from 18 to 54. We selected 18- to 22-year-olds because we were especially interested in typical 4-year college age students who are known to multi-task while studying or working [8]. Visual inspection of a histogram for age indicated 18–22 was typical college age in our population, as there was a drop in frequency from age 22 (*n* = 12) to age 23 (*n* = 2) and above. Participants were excluded if they were over 22 years of age (*n* = 84) or if they were deemed an outlier in the length of time to complete the study (*n =* 19) and the average reaction time during the task (*n* = 14). Outliers were defined as being outside 1.5 × the interquartile range (IQR). Participants were also excluded if they failed attention checks (*n* = 100) or completed the study in a social or public setting (*n* = 45). An additional 4 participants were excluded because they indicated the peg turning task did not induce boredom. Eighty-one participants reported being alone and in a quiet space and 56 participants reported being alone in a noisy space. Participants identified as White (69.30%), Hispanic/Latino (10.20%), African American/Black (1.5%), Asian (9.5%), and multiracial (9.50%). This research was approved by the Institutional Review Board and all participants provided informed consent. 

### 2.2. Design and Procedure 

Participants completed the study online using Psytoolkit [16,17]. Participants first answered demographic questions including their age, ethnicity, and their environmental conditions while participating in the study. The environmental condition question included six options: (1) alone and it is quiet, (2) alone and it is not quiet (e.g., listening to music or TV), (3) with other people but it is pretty quiet, (4) with other people and it is not quiet, (5) in a public place, and (6) somewhere else. For the two environmental conditions, only people who reported being alone in quiet and alone in not quiet environments were used to minimize social confounds. Participants then completed trait boredom scales before being randomly assigned to one of three conditions that were designed to test if strategies provided to participants influenced their ability to cope with boredom during the peg turning task. The participants then completed the peg turning task in which people turn virtual pegs one quarter turn at a time. No main effect of condition on the mean of the Multidimensional State Boredom Scale was observed, *F*(2,134) = 0.20, *p* = 0.82, so we collapsed across condition for the analyses reported herein. The peg turning task was originally developed by Festinger and Carlsmith [18] and has been rated as the most boring task out of a battery of boring tasks [13]. Participants were presented with a grid of 2 × 4 green virtual pegs and were instructed to rotate the pegs by clicking on the spacebar. Participants were only able to rotate the circles once every second. The entire task involved 10 blocks of 32 rotations (320 turns). Following the peg turning task, participants were asked to complete the Multidimensional State Boredom Scale. 

### 2.3. Measures 

#### 2.3.1. State Boredom 

State boredom was measured using the Multidimensional State Boredom Scale (MSBS) [4]. In addition to the mean of all 29 items, this scale includes 5 subscales that capture different dimensions of state boredom. These subscales are Disengagement (e.g., feelings of being stuck with nothing to do), High Arousal (e.g., impatience, irritability), Low Arousal (e.g., lethargy, fatigue), Inattention (e.g., easily distracted, difficulty focusing attention), and Time Perception (e.g., feeling that time is moving slowly/dragging on). Participants were provided with statements such as “I am stuck in a situation that I feel is irrelevant” and “Everything seems to be irritating me right now” and asked to answer how much each statement was true of how they felt during the peg turning task using a 5-point scale ranging from (1) strongly disagree to (5) strongly agree. Internal consistency for the Overall Mean Score, Disengagement, High Arousal, Low Arousal, and Time Perception all had good to excellent internal consistency (α = 0.84–0.95). The inattention subscale’s internal consistency was acceptable (α = 0.74). 

#### 2.3.2. Trait Boredom

Trait boredom was assessed using two scales. The first scale was the Short Boredom Proneness Scale (SBPS) [19]. Participants were provided with statements such as “It takes a lot of change and variety to keep me really happy” and “Much of the time, I just sit around doing nothing” and were asked to select the response that best indicated how much they agreed with the statement using a 7-point scale ranging from (1) strongly disagree to (7) strongly agree. The internal consistency was good (α = 0.86). Boredom susceptibility was measured using a modified version of the Boredom Susceptibility subscale of the Zuckerman Sensation Seeking Scale [20]. The original scale used a forced choice format to measure boredom susceptibility, which has poor internal consistency (α = 0.38–0.65) [21,22,23]. For this reason, we adapted the scale for participants to rate the original statements from the Boredom Susceptibility Scale on a 6-point scale ranging from (1) strongly disagree to (6) strongly agree. Minor updates to the statements were also made to improve clarity. For example, the statement “Looking at someone’s personal movies or videos really bores me” was changed to “Looking at a friend or family member’s personal videos or pictures (e.g., from vacations, weddings, birthdays, etc.) really bores me”. The internal consistency was acceptable (α = 0.72). 

### 2.4. Data Analysis

Data were analyzed using SPSS Statistics (Version 27, IBM). Data were first assessed for scale reliability and analysis of summary statistics (e.g., means and variances). All variables were evaluated for the presence of outliers, defined as being outside 1.5 × IQR. No outliers were present. Within the final sample, the distribution of all variables (i.e., MSBS total score and subscales, SBPS and Boredom Susceptibility Scale) were evaluated for normality using measures of skewness and kurtosis. All were found to be within a normal range defined as +/−2 [24,25]. Within the current study, skew ranged from −0.54 to 0.49 and kurtosis ranged −0.64 to −0.14. Independent samples *t*-tests were then used to test for differences in state boredom between the quiet and the noisy environments. ANCOVA was used to test for environmental influences on state boredom while controlling for the influence of trait boredom. 

## 3. Results

Results are presented in two sections. The Section 1 reports tests of the main hypothesis that boredom would be influenced by the stimulation present in their environment. The Section 2 explores the potential role of trait boredom in the environment people chose to complete the study.

**Figure 1:**
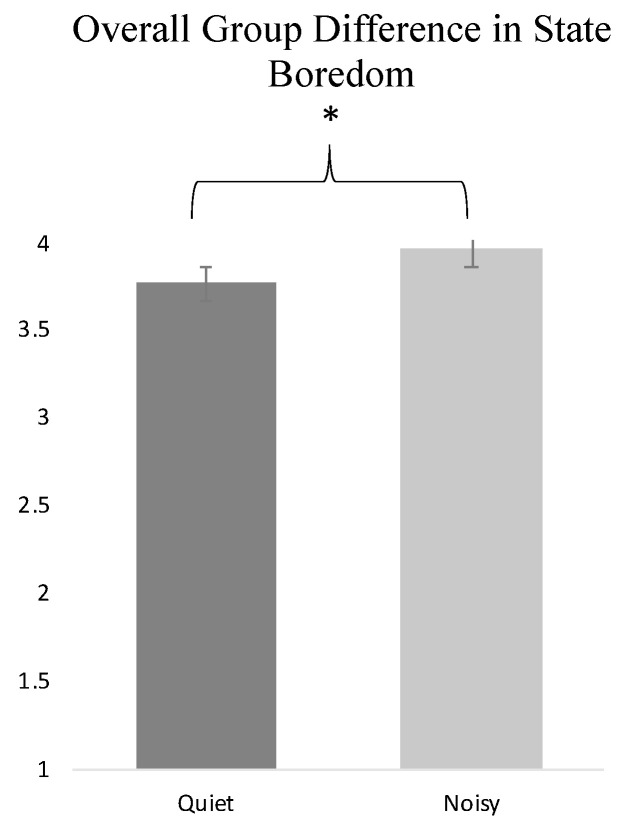
Difference in Multidimensional State Boredom Scale (MSBS) overall mean score for people in noisier and quieter environments. * *p* < 0.05. Error bars represent standard error.

**Figure 2:**
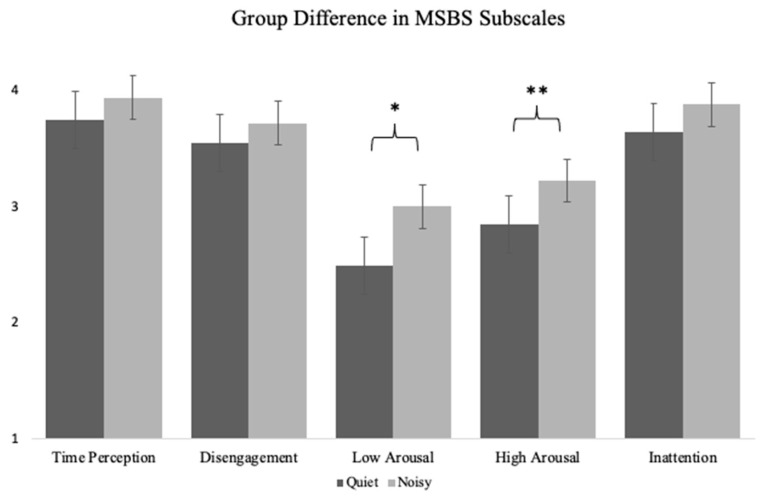
Difference in Multidimensional State Boredom Scale (MSBS) subscales for people in noisier or quieter environments. * *p* < 0.05, ** *p* < 0.01. Error bars represent standard error.

Independent samples *t*-tests were conducted to evaluate the difference between those who completed the peg turning task in a quiet or noisy environment. Results revealed that individuals who completed the peg turning task in the noisy environment had significantly higher state boredom as measured by the mean of the MSBS items, *t*(135) = −2.28, *p* = 0.02, *d* = 0.77 (see Figure 1). We wanted to explore the MSBS subscales to test whether this effect was only observed for mean MSBS, observed across all subscales, or observed on a subset of subscales. Participants in the noisy environment had higher scores on the High Arousal MSBS subscale, *t*(135) *=* −2.33, *p* = 0.02, *d* = 1.01, and Low Arousal MSBS subscale, *t*(135) *=* −2.82, *p* = 0.005, *d* = 1.08, than participants in the quiet environment (see Figure 2). There was no significant condition difference in the Disengagement, Time Perception, or Inattention subscales of the MSBS.

Individual differences in trait boredom may relate to the environments in which people complete an online study. For example, individuals high in trait boredom may seek more external stimulation when completing a mundane task. Our next set of analyses evaluated whether the effect of environment held while controlling for trait boredom. ANCOVA was used to test environmental influences on mean MSBS scores with boredom susceptibility and boredom proneness entered as covariates. Results revealed MSBS scores differed across conditions while controlling for the influence of boredom proneness and boredom susceptibility, *F*(1,133) = 4.92, *p* = 0.03, η_p_^2^ = 0.04. Boredom proneness related to mean MSBS scores, *F*(1,133) = 9.99, *p* = 0.002, η_p_^2^ = 0.07, as did boredom susceptibility, *F*(1,133) = 9.35, *p* = 0.003, η_p_^2^ = 0.07. This indicates that while trait boredom related to MSBS scores following the peg turning task, it did not account for the influence of the environment on state boredom. Using independent samples *t*-tests, we tested whether those who chose the noisy environment were higher in boredom proneness or boredom susceptibility than those who chose the quiet environment. No significant differences were observed (all *ps* > 0.10). 

## 4. Discussion

Many traditional lab-based studies were adapted to an online format at the onset of the COVID-19 pandemic. Online studies provide less experimental control over the surroundings of the participant which may have unknown influences on cognition and emotion, including boredom. We tested whether the choice to complete the peg turning task as part of an online study in a quiet or noisy environment influenced state boredom. We found that individuals who were in a noisier environment experienced more boredom than those in a quieter environment, even after controlling for the influence of trait boredom. Prior studies have shown college students often turn to social media or music while studying, motivated in part by the desire to pass time [8]. Perhaps unintuitively, this practice may result in more boredom. The current study raises theoretically interesting questions about why a noisy environment influences boredom and holds important implications for both conducting online studies and mitigating boredom in real world settings.

Theories of boredom shed some light on the potential mechanisms at work in quiet, relative to noisy environments. The MAC model posits that boredom arises when task demands are insufficient to fully engage attention or are too difficult and exceed attentional capacity [7]. In support of the MAC model, Westgate and Wilson [7] found that participants reported higher levels of boredom while completing a task designed to be under-stimulating relative to a variant of the same task designed to optimally engage attention. Additionally, they found a u-shaped relationship between self-report of task difficulty and boredom such that both lower and higher levels of difficulty related to higher levels of boredom. This pattern of results is consistent with the idea boredom arises when attentional demands are too low or too high. It is possible the combination of completing the peg turning task and noise from the environment was too stimulating, dividing and taxing attention, leading to boredom. Westgate and Wilson [7] simply asked people to rate boredom on a Likert scale. We used the MSBS, which provides more fine-grained information about experience across dimensions that contribute to boredom, including attentional engagement and affect. Our results did not reveal a condition effect on the inattention subscale of the MSBS, which measures distraction and difficulty paying attention. We did find that people in more noisy environments reported feeling more highly aroused and agitated (High Arousal subscale) as well as more lonely and down (Low Arousal subscale), two important pieces of state boredom [4]. These findings might indicate the noisy environment created an expectation for engagement that was not met, leading to both agitation and feelings of loneliness. This interpretation fits well with the Dual-Self model, which posits that environmental cues set expectations for engagement that, when unmet, result in boredom [10]. It is possible that environments with TV and music are associated with engagement, but when combined with the need to complete a tedious task, results in negative feelings. 

Our results also fit with opportunity cost models of boredom, which view the value of allocating resources and effort relative to alternatives [10,26]. When more optimally engaging alternatives are present, the cost of completing a less engaging task induces unpleasant feelings, signaling a need to shift toward a more engaging alternative. Struk et al. [27] tested the opportunity cost explanation of boredom in a lab setting by asking people to sit in a room for 15 min alone under conditions with or without objects present. Participants in both conditions were told to refrain from engaging with the objects and entertain themselves. Participants reported higher levels of boredom when objects were present than when they were not, consistent with the idea more engaging alternatives increased the unpleasant feeling of boredom. When the results of the current study are viewed within an opportunity cost framework, the noisy environment indicates that more engaging alternatives are available, which, in turn, increases unpleasant feelings, such as boredom. Importantly, we do not know why some people completed the study in a noisy environment. It may have been incidental or purposeful. 

An important contribution of the current study is evidence of the consistency across both lab and real-world settings of experiencing boredom when potentially more engaging alternatives are present [27]. Historically, cognition, emotion, and behavior have been studied in controlled lab settings. Online studies on these topics, including boredom, rose during the pandemic and may continue as a popular method for data collection. Therefore, the results from this study include important considerations for online data collection. Online data collection provides relatively easier access to a range of participants for research studies than traditional lab-based studies. It is widely used and even more so during the COVID-19 pandemic when in-person data collection was largely prohibited. Our data indicated that the environmental conditions in which participants completed an online study impacts their emotional experience, which might, in turn, impact results. One methodological implication for future studies collected outside a controlled lab environment is to ask questions about the environmental conditions. 

The results of the current study might have implications for people’s experience while completing school and work tasks remotely. Recent research found that many employees working remotely during the COVID-19 pandemic attributed increased environmental flexibility to improved well-being [28]. Some children described improvements in environmental conditions when attending school remotely during the COVID-19 pandemic; however, many children also reported feeling bored frequently in remote learning environments [29]. While the initial shift to remote work and school in 2020 was due to safety measures in place to reduce the spread of COVID-19, remote options have remained prominent even with relaxed pandemic restrictions [30]. The environment in which people choose to complete work and school tasks might influence how bored, agitated, or under-aroused they feel. Workplaces and schools might encourage their employees and students to minimize stimulation external to the task at hand. The results of the current study and similar studies [27] might motivate remote workers, learners, employers, and teachers to investigate the impact of environmental conditions on productivity, well-being, and learning. Further research is needed to understand the impact of environmental noise and stimulation on boredom in relation to remote work and school.

An important limitation of our study is that the environment participants were in while completing the study was not experimentally manipulated. There are several future research directions that can help us better understand the role of environment on the experience of boredom. In the current study, environmental conditions were self-reported. We do not have information on actual noise present in the environment except it was likely TV or music, as these elements were part of our question. Thus, one question for future research is what combination of tasks and types and quantity of noise in the environment influences boredom. Within an opportunity cost framework, the noise needs to indicate relatively more engagement than the task at hand. From this perspective, the presence of TV, conversation, or music may induce more boredom than simply white noise. Testing these possibilities is important to identify strategies to modify the environment to mitigate boredom.

In conclusion, the COVID-19 pandemic prompted many researchers to adapt lab-based studies to an online format, which offers less experimenter control over the environment. We found individuals who completed a mundane task as part of an online study in a noisier environment experienced more boredom than those who completed the study in a quieter environment. Our study showed that individuals who completed a mundane task as part of an online study in a noisier environment experienced more boredom than those who completed the study in a quieter environment. This may have occurred because noisier environments divide attention, set expectations for engagement that go unmet, or indicate a more interesting alternative is available. Importantly, we cannot test between these possibilities, and they may not be mutually exclusive alternatives. This study has important methodological implications. Many researchers routinely use online data collection methods. Our results indicate that the environmental conditions people choose to complete studies in impacts how they feel, which might impact performance. Since controlling the environment participants complete studies in is difficult, asking participants to describe and report on their environment allows researchers to better understand these influences. This study also holds implications for real-world boredom reduction strategies. This study suggests that reducing outside stimulation and focusing only on the task at hand will result in less boredom. This may be especially important for students or employees who might be required to persist through tedious tasks.

## Data Availability

The data presented in this study are available in supplementary material (submitted upon article acceptance).

## Supplementary Materials

The following supporting information can be downloaded at https://www.mdpi.com/article/10.3390/bs12080282/s1.
